# The Adsorption Mechanisms of SF_6_-Decomposed Species on Tc- and Ru-Embedded Phthalocyanine Surfaces: A Density Functional Theory Study

**DOI:** 10.3390/molecules28207137

**Published:** 2023-10-17

**Authors:** Rou Xue, Wen Jiang, Xing He, Huihui Xiong, Gang Xie, Zhifeng Nie

**Affiliations:** 1 Yunnan Key Laboratory of Metal-Organic Molecular Materials and Device, School of Chemistry and Chemical Engineering, Kunming University, Kunming 650214, China; xuer682@163.com (R.X.); hx036044@163.com (X.H.); 2 School of Metallurgy Engineering, Jiangxi University of Science and Technology, Ganzhou 341000, China; xionghui8888@126.com; 3 Kunming Metallurgical Research Institute Co., Ltd., Kunming 650031, China; gangxie@sina.com

**Keywords:** adsorption mechanism, metal phthalocyanine monolayer, SF_6_-decomposed species, density functional theory

## Abstract

Designing high-performance materials for the detection or removal of toxic decomposition gases of sulfur hexafluoride is crucial for both environmental monitoring and human health preservation. Based on first-principles calculations, the adsorption performance and gas-sensing properties of unsubstituted phthalocyanine (H_2_Pc) and H_2_Pc doped with 4d transition metal atoms (TM = Tc and Ru) towards five characteristic decomposition components (HF, H_2_S, SO_2_, SOF_2_, and SO_2_F_2_) were simulated. The findings indicate that both the TcPc and RuPc monolayers are thermodynamically and dynamically stable. The analysis of the adsorption energy indicates that H_2_S, SO_2_, SOF_2_, and SO_2_F_2_ underwent chemisorption on the TcPc monolayer. Conversely, the HF molecules were physisorbed through interactions with H atoms. The chemical adsorption of H_2_S, SO_2_, and SOF_2_ occurred on the RuPc monolayer, while the physical adsorption of HF and SO_2_F_2_ molecules was observed. Moreover, the microcosmic mechanism of the gas–adsorbent interaction was elucidated by analyzing the charge density differences, electron density distributions, Hirshfeld charges, and density of states. The TcPc and RuPc monolayers exhibited excellent sensitivity towards H_2_S, SO_2_, and SOF_2_, as evidenced by the substantial alterations in the band gaps and work functions of the TcPc and RuPc nanosheets. Our calculations hold significant value for exploring the potential chemical sensing applications of TcPc and RuPc monolayers in gas sensing, with a specific focus on detecting sulfur hexafluoride.

## 1. Introduction

Sulfur hexafluoride (SF_6_) is a non-flammable gas with outstanding insulating medium properties and quenching arc performance [1]. Due to its robust dielectric properties, relatively low toxicity, and exceptional chemical inertness, SF_6_ is frequently employed in diverse industrial applications and processes [2,3,4]. During prolonged equipment operation, insulation defects are inevitable and partial discharges take place, leading to the decomposition of SF_6_ and the production of harmful fluoride sulfides. When trace amounts of water and oxygen are present, significant reactions occur with these by-products, resulting in the formation of gaseous substances, including hydrogen fluoride (HF), hydrogen sulfide (H_2_S), sulfur dioxide (SO_2_), thionyl fluoride (SOF_2_), and sulfuryl fluoride (SO_2_F_2_) [5,6,7]. Collectively, these substances are referred to as the characteristic components of SF_6_. These characteristic components not only affect the insulation performance of electrical equipment and corrode solid insulation materials [8,9], but also pose a risk to human health, such as causing eye/skin irritations, allergies, and even cancers [10,11]. Therefore, the online detection and scavenging of SF_6_-decomposed gases has become an urgent task for evaluating the safety of gas-insulated substation (GIS) operation and safeguarding human well-being [12,13,14].

More recently, two dimensional (2D) phthalocyanine (Pc) nanomaterials, which were successfully synthesized using an experimental method, have received widespread attention due to their unique properties [15,16,17]. Interestingly, phthalocyanine displays a variety of morphologies and possesses exceptional properties such as a high specific surface area, unique electronic characteristics, and attractive optical performance [18,19]. The exceptional properties exhibited by phthalocyanine make it a highly valuable material for advanced technical applications, including photovoltaics [20,21], optoelectronics [22,23], electrocatalysis [24,25], and spintronics [26,27]. Furthermore, a subset of phthalocyanine materials, which operate at ambient temperatures, is both safe and non-hazardous, demonstrating significant potential for integration into wearable technology [28,29]. With the remarkable advancements in preparation methods, including organic vapor deposition (OVPD) [30], chemical synthesis [31], and epitaxial growth [32], the production of phthalocyanine materials with high yields and large surface areas has significantly improved, which has led to phthalocyanine-based nanomaterials being extensively used in resistance, optical, and micro-weight precision sensors [33,34,35]. However, their limited sensitivity and selectivity, as well as their slow sensing processes, impede their practical application [36].

Currently, the decoration of transition metal (TM) is considered an effective method for enhancing the sensing performance of 2D materials. Similarly, the semiconductor properties and sensing abilities of H_2_Pc can be altered by introducing a TM into the center of the H_2_Pc monolayer [37,38,39]. For instance, incorporating Cr into pristine phthalocyanine has been shown to improve its selectivity and sensitivity towards H_2_CO [40]. With the aid of DFT computations, Zou et al. discovered that MnPc exhibited excellent sensing abilities towards CO, NO, O_2_, and NO_2_ by examining the adsorption behaviors and sensing characteristics of six different gas molecules on an MnPc monolayer [41]. Furthermore, various TM-atom-modified phthalocyanine monolayers, such as CoPc [42,43,44], FePc [45], CuPc [42], and NiPc [42,46], have demonstrated exceptional gas-sensing properties. The outstanding performance achieved with 3d TM atoms doped into an H_2_Pc monolayer raises the following question: can 4d TM atoms, when similarly doped into an H_2_Pc monolayer, serve as exceptional gas sensors or adsorbents for detecting or scavenging SF_6_ decomposition species?

In this work, we propose TMPc (TM = Tc and Ru) nanomaterials as a possible gas-sensing material for the detection of SF_6_ decomposition products based on the first-principles calculations. First of all, the adsorption performance of five SF_6_ decomposition gases (HF, H_2_S, SO_2_, SOF_2_, and SO_2_F_2_) on TcPc and RuPc monolayers are systematically studied. Then, the electronic characteristics of the different adsorption systems are computed and analyzed, including the charge transfer, charge density differences (CDDs), electron density distributions (EDDs), density of states (DOSs), and partial density of states (PDOSs). Subsequently, we discuss the gas-sensing mechanisms of TcPc and RuPc towards these toxic gases by examining changes in band structures and work functions before and after gas adsorption. Finally, the feasibility of employing TcPc and RuPc monolayers as promising materials for detecting SF_6_ characteristic components is assessed based on their adsorption strengths and sensing performances.

## 2. Results and Discussion

### 2.1. Structure and Stability of H_2_Pc, TcPc, RuPc, and SF_6_

**Figure 1:**
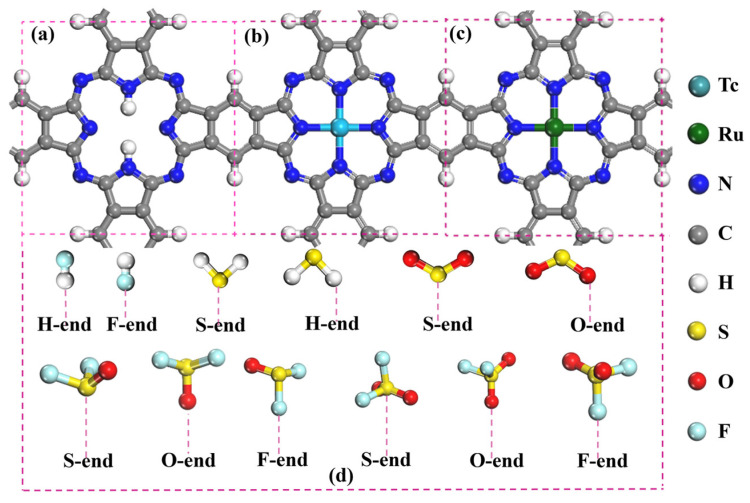
Top sides of the optimized geometric structures: (**a**) H_2_Pc, (**b**) TcPc, (**c**) RuPc, and (**d**) various adsorption styles of SF_6_-decomposed species. The cyan, green, blue, gray, white, yellow, red, and light blue balls are Tc, Ru, N, C, H, S, O, and F atoms, respectively.

In the TMPc (TM = Tc and Ru) monolayer, as displayed in Figure 1b,c, the Tc/Ru atom is surrounded by four nearest-neighbor N atoms (N4) in the center vacancy, and the suspended bonds of C atoms are passivated with the H atoms. Moreover, all atoms in the three optimized geometric structures are coplanar. According to our previous investigation [47,48], the hollow site of the H_2_Pc and TMPc monolayer is the preferential adsorption site for the “porphyrin-like” configurations. When the SF_6_ decomposition components approach the H_2_Pc/TMPc monolayers, various adsorption styles of SF_6_ decomposition gases are considered, as shown in Figure 1d. For instance, two different adsorption configurations of HF on the H_2_Pc/TMPc monolayers are considered: HF adsorption on the substrate with the H atom orients towards the surface (defined as the H-end) and HF adsorption on the substrate with the F atom orients towards the surface (defined as the F-end).

**Figure 2:**
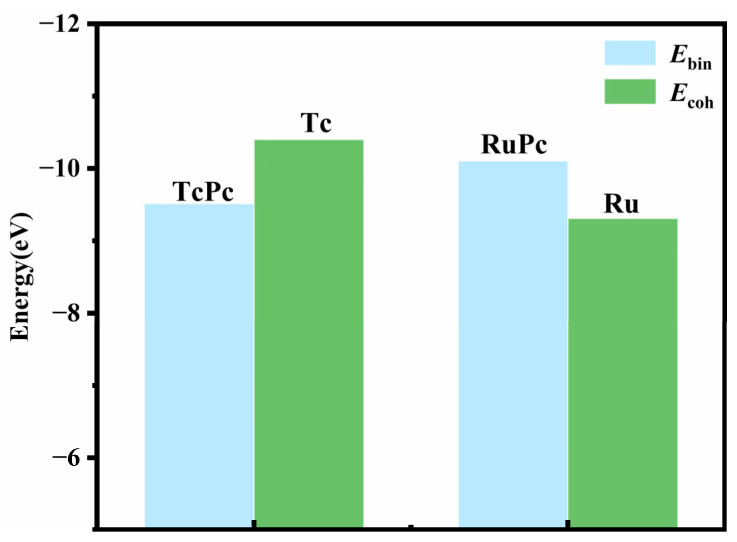
Binding energy (*E*_bin_) of TcPc/RuPc monolayer and cohesive energy (*E*_coh_) of Tc/Ru bulk.

**Figure 3:**
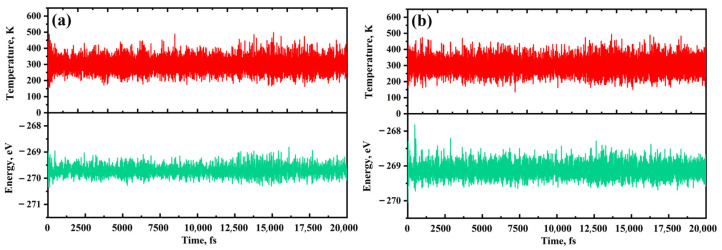
Temperature and potential energy fluctuation of (**a**) TcPc and (**b**) RuPc monolayers in the AIMD simulation at 300 K. The red and green lines correspond to the temperature and potential energy fluctuations, respectively.

Figure 2 illustrates that the binding energy (*E*_bin_) of RuPc is smaller than the cohesive energy (*E*_coh_) of bulk Ru, indicating robust thermal stability in the RuPc monolayer [47]. Despite TcPc having a larger *E*_bin_ than the *E*_coh_ of bulk Tc, the negative value of the binding energy is sufficient to maintain the structural stability of the TcPc monolayer [48]. The high stability of both the RuPc and TcPc monolayers is further supported by an ab initio molecular dynamics (AIMD) simulation, as depicted in Figure 3. Here, the temperature and potential energy of TcPc and RuPc exhibit only slight fluctuations around the equilibrium state after heating at 300 K for 20 ps with a time step of 1 fs. Therefore, both the TcPc and RuPc monolayers exhibit sufficient stability for application as sensing materials.

**Figure 4:**
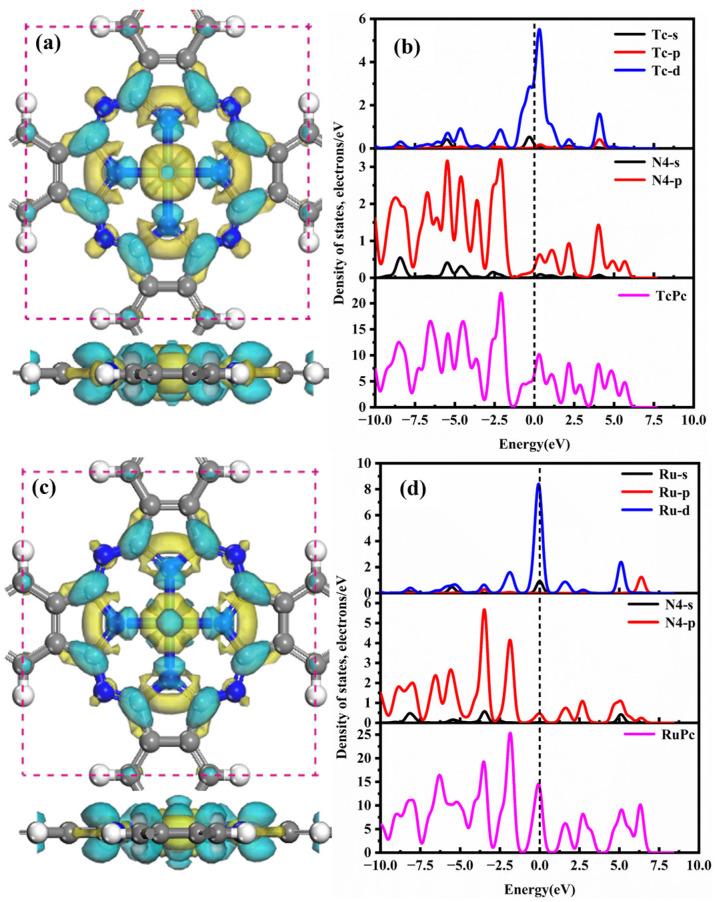
CDD and DOSs of the (**a**,**b**) TcPc and (**c**,**d**) RuPc monolayer. The four nearest-neighbor N atoms of TcPc/RuPc monolayer are indicated by N4. The cyan and yellow areas correspond to the charge accumulation and consumption, respectively. The isosurface of CDD is set as ±0.01 eÅ^−3^, and the black dashed line is the Fermi level.

Figure 4 depicts the CDD and DOSs of the TcPc and RuPc monolayers. In Figure 4a, the Tc atom donates 0.213 e, acting as an electron donor, while its nearest-neighbor N4 atoms accept 0.068 e, functioning as electron acceptors. Additionally, charge accumulation primarily occurs on the C and N atoms, while charge depletion surrounds the central Tc atom, highlighting the strong affinity between the Tc atom and N4 atoms. As shown in Figure 4b, the robust interactions between Tc and the Pc monolayer are predominantly due to the strong hybridizations of Tc-d and N-p orbitals in the range of −5.0 to 5.0 eV. Consequently, the pronounced affinity between Tc and Pc contributes to the robust stability of the TcPc monolayer. From Figure 4c–d, it is evident that the CDD and DOS plots of the RuPc nanosheet share similarities with those of the TcPc monolayer. The central Ru atom also acts as an electron donor, interacting with the N4 atoms across a wide energy range of −7.5 to 7.5 eV. Notably, at the Fermi level, there is an overlap between the Ru-sp orbital and the p orbital of the N4 atom, resulting in the formation of a strong Ru-N4 bond.

### 2.2. Adsorption Characteristics of SF_6_ Decomposition Gases on H_2_Pc Monolayer

In this section, the adsorption of bare H_2_Pc for five characteristic decomposed species of SF_6_ was first analyzed. The corresponding calculated data, including adsorption energy (*E*_ads_), adsorption distance (*D*), electron transfer (*Q*_t_) and bandgap (*B*_g_), are listed in Table 1. Table 1 shows that the SF_6_-decomposed gas molecules, except for HF, prefer to adsorb onto the H_2_Pc substrate via the S-end. For different adsorption systems, the adsorption energy follows the order: H_2_S < HF < SO_2_F_2_< SOF_2_ < SO_2_. Meanwhile, all of the absolute values of adsorption energy are below the critical value of 0.8 eV [49]; thus, the adsorption process can be classified as physisorption, which is mainly contributed by the van der Waals interaction [50]. In addition, the five characteristic species have little effect on the energy gap of pristine H_2_Pc. Therefore, the pure H_2_Pc monolayer is not suitable to detect and remove SF_6_ decomposition species due to the poor sensitivity and weak adsorption strength.

**Figure 5:**
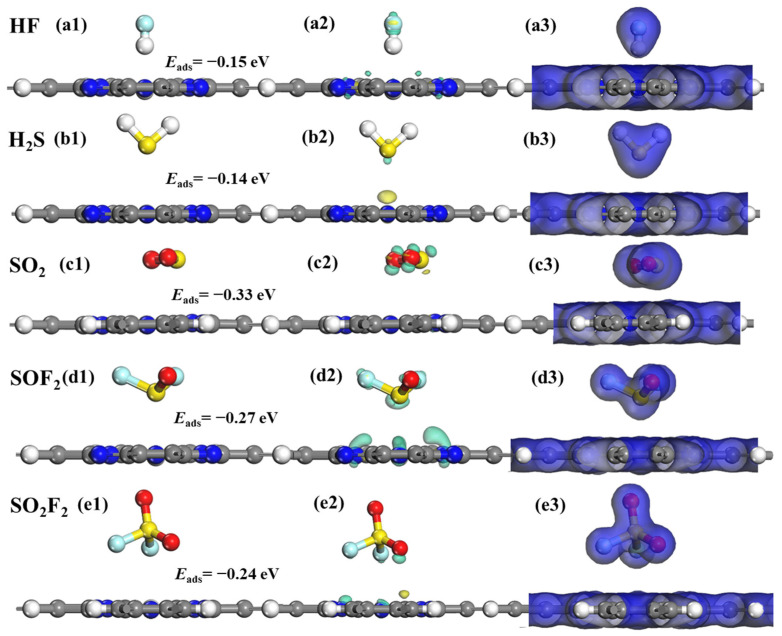
Optimized geometric models, CDD, and EDD of SF_6_-decomposed species adsorption on H_2_Pc monolayer. (**a1**–**a3**) HF, (**b1**–**b3**) H_2_S, (**c1**–**c3**) SO_2_, (**d1**–**d3**) SOF_2_, and (**e1**–**e3**) SO_2_F_2_. The cyan and yellow areas correspond to the accumulation and consumption of charges, respectively. The isosurface values of CDD and EDD are set as ±0.003 eÅ^−3^ and 0.2 eÅ^−3^, respectively.

Figure 5 shows the lowest-energy CDD and EDD of different adsorption systems. One can see that the SF_6_ decomposition component has a negligible impact on the structure of the H_2_Pc monolayer, with the coplanar structures of H_2_Pc remaining intact after the adsorption of these gases. Furthermore, as observed in the CDD plots of Figure 5b2–e2, only a minimal amount of electron transfer occurs in the HF@H_2_Pc and H_2_S@H_2_Pc adsorption systems. This suggests that the interactions between HF, H_2_S, and the H_2_Pc substrate are relatively weaker compared to the other three adsorption systems. Simultaneously, as demonstrated in the EDD plots of Figure 5b3–e3, the electron densities of all SF_6_ decomposition products do not overlap with those of the H_2_Pc substrate, further indicating that their interactions are not particularly strong. Those results are in good agreement with the calculations listed in Table 1. Consequently, the pristine H_2_Pc nanosheet cannot become a potential sensing material for SF_6_-decomposed species in terms of adsorption strength and electron transfer.

### 2.3. Adsorption Characteristics of SF_6_ Decomposition Gases on TcPc Monolayer

The preferential adsorption orientation, adsorption energy, adsorption distance, electron transfer, and bandgap of the SF_6_ decomposition components on TcPc monolayers are summarized in Table 2. It is found that the HF species prefers to adsorb onto the TcPc nanosheet with the H-end, whereas the H_2_S, SO_2_, SOF_2_ and SO_2_F_2_ molecules prefer to adsorb on TcPc monolayer with the S-end. Moreover, except for the HF one, the other four decomposed species are chemically adsorbed with *E*_ads_ of −1.43 eV, −1.97 eV, −1.78 eV, and −0.96 eV, respectively. Meanwhile, an electron transfer of 0.298 e, 0.099 e, 0.073 e, and 0.256 e occurs between the corresponding gas molecules and the TcPc substrate. Interestingly, there is a clear variation in the bandgap of TcPc after the adsorption of H_2_S, SO_2_, and SOF_2_ molecules, showing a potential sensitivity of the TcPc monolayer towards these three species.

**Figure 6:**
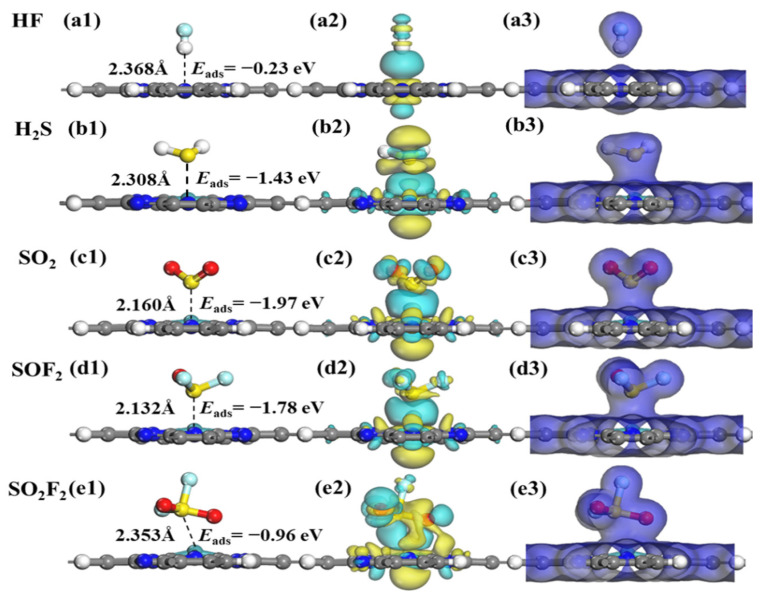
Optimized geometric models, CDD, and EDD of SF_6_-decomposed species adsorption on TcPc monolayer. (**a1**–**a3**) HF, (**b1**–**b3**) H_2_S, (**c1**–**c3**) SO_2_, (**d1**–**d3**) SOF_2_, and (**e1**–**e3**) SO_2_F_2_. The cyan and yellow areas correspond to the charge accumulation and consumption, respectively. The isosurface values of CDD and EDD are set as ±0.01 eÅ^−3^ and 0.2 eÅ^−3^, respectively.

The optimized structures, CDD, and EDD of SF_6_-decomposed species adsorption on TcPc monolayer are presented in Figure 6. In Figure 6a1, the HF molecule is adsorbed on the TcPc nanosheet with an adsorption energy of −0.23 eV and an adsorption distance of 2.368 Å. The adsorption energy of HF@TcPc falls below the critical threshold of 0.8 eV, suggesting that it belongs to a physical adsorption driven by van der Waals forces. In contrast, as shown in Figure 6b1–e1, the chemisorption occurs among the other four adsorption systems with *E*_ads_ ranging from −0.96 to −1.97 eV. In addition, as shown in Figure 6a2–e2, there is very little electron transfer in the HF@TcPc adsorption system, indicating that the interactions between HF molecules and the TcPc nanosheet are much weaker compared to the other four adsorption systems. For the SO_2_@TcPc, SO_2_F@TcPc, and SO_2_F_2_@TcPc systems, abundant charges are accumulated around the gas molecules, whereas some charges are depleted around the corresponding TcPc monolayer. These results suggest that massive electrons from the TcPc nanosheet are transferred to the SO_2_, SO_2_F, and SO_2_F_2_. Furthermore, there is substantial overlap in the total electron density between H_2_S, SO_2_, SO_2_F_2_, SO_2_F_2_, and TcPc substrate, as shown in the EDD maps in Figure 6a3–e3. This further demonstrates that the TcPc monolayer possesses a strong trapping capability for H_2_S, SO_2_, SOF_2_, and SO_2_F_2_ molecules.

**Figure 7:**
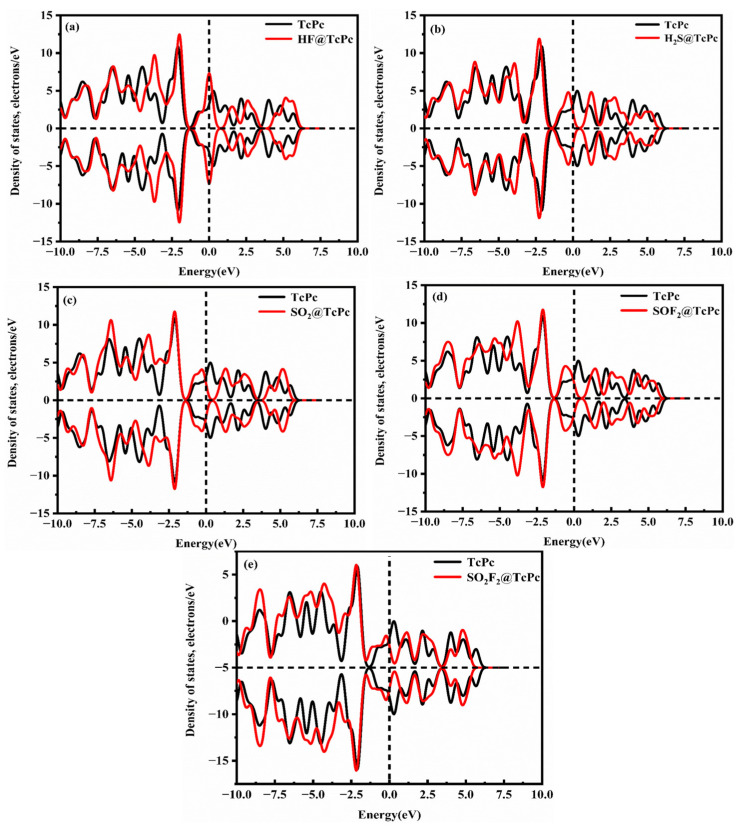
DOSs of different gas@TcPc adsorption systems. (**a**) HF@TcPc, (**b**) H_2_S@TcPc, (**c**) SO_2_@TcPc, (**d**) SOF_2_@TcPc, and (**e**) SO_2_F_2_@TcPc. The black and red lines represent the DOSs of the TcPc monolayer before and after gas adsorption, respectively. The Fermi level serves as the zero-energy reference point and is represented by a vertical black dashed line.

**Figure 8:**
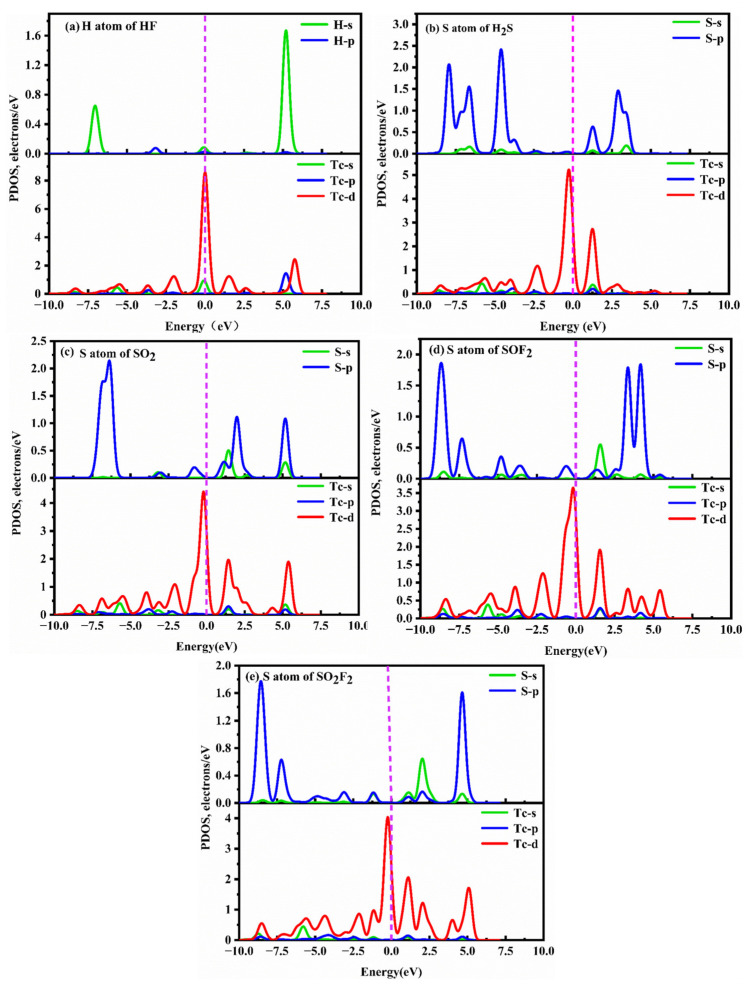
PDOSs of different gas@TcPc adsorption systems. (**a**) HF@TcPc, (**b**) H_2_S@TcPc, (**c**) SO_2_@TcPc, (**d**) SOF_2_@TcPc, and (**e**) SO_2_F_2_@TcPc. The Fermi level is indicated by the vertical dashed line at zero energy.

To gain a better understanding of the adsorption behavior of five SF_6_ decomposition products on the TcPc substrate, the corresponding DOSs and PDOSs of different adsorption systems before and after gas adsorption are presented in Figure 7 and Figure 8. Figure 7 illustrates the symmetric DOSs of the five adsorption systems, indicating their non-magnetic nature [51]. The data in Figure 7 make it evident that, unlike the other four systems, the HF system has a minimal impact on the DOSs near 0 eV. Additionally, there are no discernible resonance peaks between H-p and Tc-d orbits (Figure 8a). Upon adsorption of H_2_S, SO_2_, SOF_2_, and SO_2_F_2_ onto TcPc in the respective systems, the DOS curve shows a slight shift towards lower energy levels (Figure 7b–e). Notably, a distinct peak emerges near the Fermi level, indicating a significant transfer of electrons from the TcPc surface to the H_2_S, SO_2_, SOF_2_, and SO_2_F_2_ molecules. This leads to strong interactions between the gas and substrate. The notable adsorption strength is primarily attributed to the orbital hybridization between S-sp and Tc-d orbitals across the entire energy level, as illustrated in Figure 8. However, in the HF@TcPc system, there are four weak resonance peaks at approximately −6.11 eV, −0.23 eV, 2.32 eV, and 5.10 eV. This suggests that the TcPc nanosheet has a limited capacity for trapping HF. Therefore, the TcPc monolayer demonstrates significant potential as a sensing material for H_2_S, SO_2_, SOF_2_, and SO_2_F_2_ molecules.

### 2.4. Adsorption Characteristics of SF_6_ Decomposition Gases on RuPc Monolayer

Similarly, the adsorption properties of gas molecules including HF, H_2_S, SO_2_, SOF_2_, and SO_2_F_2_ on the RuPc monolayers were also investigated thoroughly. The adsorption energy, electron transfer, adsorption distance, and band structure are listed in Table 3. The adsorption energies of HF, H_2_S, SO_2_, SOF_2_, and SO_2_F_2_ molecules are −0.28 eV, −1.26 eV, −1.64 eV, −1.53 eV, and −0.33 eV, respectively. The corresponding adsorption distances between HF, H_2_S, SO_2_, SOF_2_, SO_2_F_2_, and RuPc surface are 2.268 Å, 2.243 Å, 2.110 Å, 2.088 Å, and 3.411 Å, respectively. In terms of adsorption energies, the RuPc sheet exhibits strong trapping capabilities for H_2_S, SO_2_, and SOF_2_ molecules. However, the trapping efficiency for HF and SO_2_F_2_ is relatively limited. Furthermore, approximately 0.165 e, 0.071 e, and 0.039 e are transferred from the RuPc nanosheet to the HF, SO_2_, and SOF_2_ molecules, respectively. Conversely, the RuPc sheet received approximately 0.275 e and 0.003 e from the H_2_S and SO_2_F_2_ molecules, respectively. This indicates that HF, SO_2_, and SOF_2_ (H_2_S and SO_2_F_2_) function as electron acceptors (donors). Consequently, the RuPc monolayer possesses a suitable adsorption capacity for H_2_S, SO_2_, and SOF_2_ molecules, indicating its potential as a gas sensor material. Additionally, the band structure of RuPc undergoes noticeable alterations upon the adsorption of H_2_S, SO_2_, and SOF_2_, further emphasizing its remarkable gas sensitivity towards these molecules.

**Figure 9:**
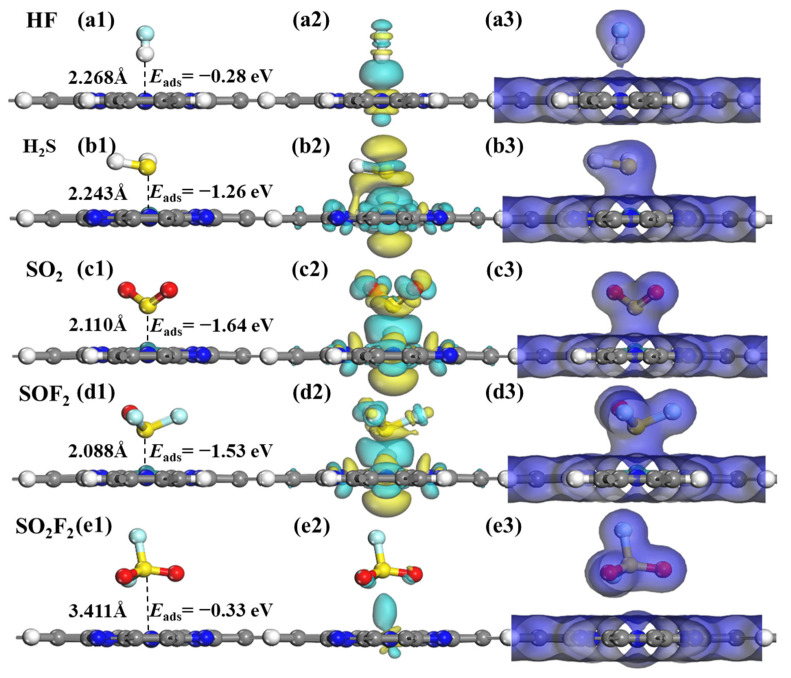
Optimized geometric models, CDD, and EDD of SF_6_-decomposed species adsorption on RuPc monolayer. (**a1**–**a3**) HF, (**b1**–**b3**) H_2_S, (**c1**–**c3**) SO_2_, (**d1**–**d3**) SOF_2_, and (**e1**–**e3**) SO_2_F_2_. The cyan and yellow areas correspond to the charge accumulation and consumption, respectively. The isosurface values of CDD and EDD are set as ± 0.01 eÅ^−3^ and 0.2 eÅ^−3^, respectively.

Figure 9 illustrates the lowest energy structures, CDD, and EDD of the SF_6_ decomposition gas adsorbed on the RuPc monolayer. As displayed in Figure 9a1–d1, a clear trend can be observed in the adsorption energies (*E*_ads_), as follows: *E*_ads_ (HF) > *E*_ads_ (SO_2_F_2_) > *E*_ads_ (H_2_S) > *E*_ads_ (SOF_2_) > *E*_ads_ (SO_2_). In general, adsorption processes with *E*_ads_ greater than 0.8 eV are commonly classified as chemisorption [52], and the adsorption energies of H_2_S, SO_2_, and SOF_2_ molecules on the RuPc monolayer are −1.26 eV, −1.64 eV, and −1.53 eV, respectively. Obviously, the RuPc monolayer exhibits appropriate adsorption strength to chemically capture H_2_S, SO_2_, and SOF_2_ gas molecules. In contrast, the interaction between HF, SO_2_F_2_ molecules, and the RuPc monolayer is weak. The adsorption energies of HF and SO_2_F_2_ are −0.28 eV and −0.33 eV, with corresponding adsorption distances of 2.268 Å and 3.411 Å, respectively. This outcome indicates that HF and SO_2_F_2_ weakly adsorb onto the RuPc monolayer, primarily driven by van der Waals forces. The CDD plots in Figure 9a2–e2 demonstrate electron transfer occurring between H_2_S, SO_2_, SOF_2_, and the RuPc nanosheet. From Figure 9a2 and e2, the presence of large electron depletion between HF/SO_2_F_2_ and RuPc also demonstrates their weak interaction. However, abundant electrons of H_2_S/SO_2_/SOF_2_ are transferred to the intermediate region of the gas–substrate, resulting in a strong interaction between H_2_S/SO_2_/SOF_2_ and the RuPc monolayer. In addition, HF, SO_2_, and SOF_2_ acquire a few electrons from the RuPc monolayer, acting as electron acceptors. Conversely, the other gas molecules function as electron donors, releasing a portion of their electrons. This observation aligns well with the results obtained from Hirshfeld charge analysis (Table 3). As shown in Figure 9a3–e3, there exists a significant electron overlap between H_2_S, SO_2_, and SOF_2_ molecules and the RuPc monolayer, while electron overlap does not occur in the HF@RuPc and SO_2_F_2_@RuPc systems. Consequently, it is concluded that the RuPc monolayer has a strong capture ability in terms of H_2_S, SO_2_, and SOF_2_ molecules.

**Figure 10:**
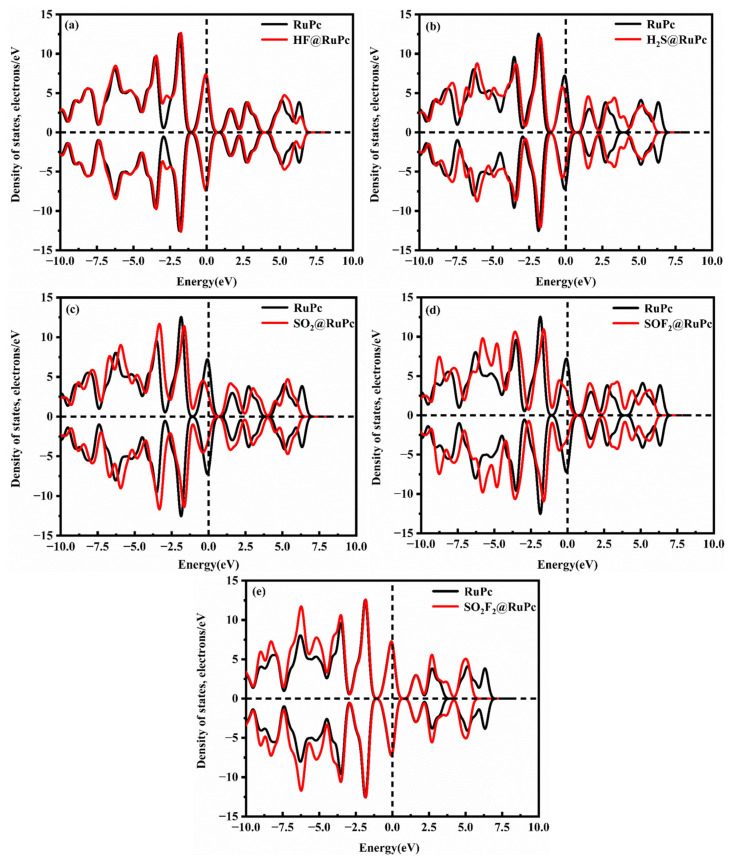
DOSs of different gas@RuPc adsorption systems. (**a**) HF@RuPc, (**b**) H_2_S@RuPc, (**c**) SO_2_@RuPc, (**d**) SOF_2_@RuPc, and (**e**) SO_2_F_2_@RuPc. The black and red lines represent the DOSs of the RuPc monolayer before and after gas adsorption, respectively. The Fermi level serves as the zero-energy reference point and is represented by a vertical black dashed line.

**Figure 11:**
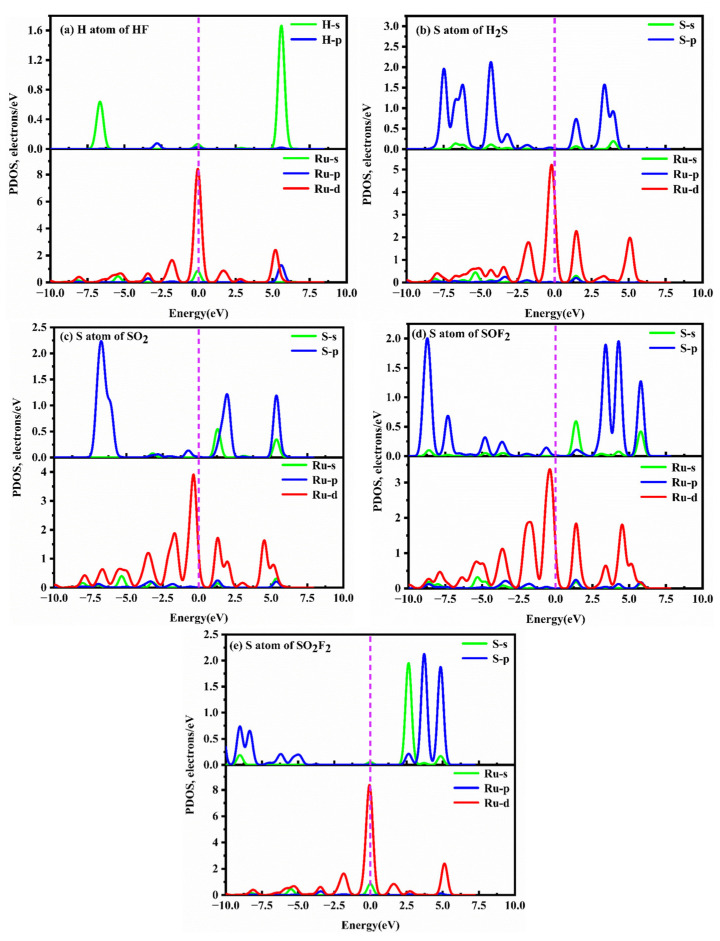
PDOSs of different gas@RuPc adsorption systems. (**a**) HF@RuPc, (**b**) H_2_S@RuPc, (**c**) SO_2_@RuPc, (**d**) SOF_2_@RuPc, and (**e**) SO_2_F_2_@RuPc. The Fermi level is indicated by the vertical dashed line at zero energy.

To further understand the microcosmic mechanism of the gas–substrate interaction, the DOSs and PDOSs of SF_6_ decomposition species on the RuPc monolayer are displayed in Figure 10 and Figure 11. From Figure 10a, it is evident that the DOSs of the RuPc monolayer with and without HF adsorption are nearly identical in the energy interval of −2.40 to 5.00 eV and −2.75 to 2.45 eV. When compared to the clean RuPc monolayer, the DOSs of the HF@RuPc system experience a slight increase after 5.0 eV and 2.45 eV, with peaks appearing at around −2.00 eV and 0.00 eV, and no significant shift observed. The unoccupied DOS peak of this system is primarily contributed by the Ru-d orbital and H-s orbital. Moreover, there is no state peak overlap between the two atoms near the Fermi level (Figure 11a); thus, the interaction is extremely weak. As shown in Figure 10b, the DOSs of clean RuPc and H_2_S@RuPc near the Fermi level are different: the DOS peak value of H_2_S@RuPc moves to the low-energy direction. From Figure 11b, the interaction between the S atom and Ru atom is evident due to the obvious resonance peaks between the S and Ru atoms at about 2.00 eV. Combining the *E*_ads_ (−1.26 eV) and electron transfer (0.275 e) of the H_2_S@RuPc system, one can conclude that the adsorption of H_2_S on the RuPc monolayer belongs to chemical adsorption. The DOSs of the SO_2_ and SOF_2_ adsorption systems are similar to that of H_2_S, as shown in Figure 10c,d. Both of them have deviations in DOSs at the Fermi level, and their peaks are reduced. From Figure 11c,d, resonance peaks of S and Ru atoms near the Fermi level can be observed, indicating that strong interactions of SO_2_, SOF_2_, and the RuPc monolayer exist in the two systems. In conclusion, the RuPc monolayer can be used as a candidate sensing material for the detection of H_2_S, SO_2_, and SOF_2_.

### 2.5. Sensing Performance Evaluation of TcPc and RuPc

**Figure 12:**
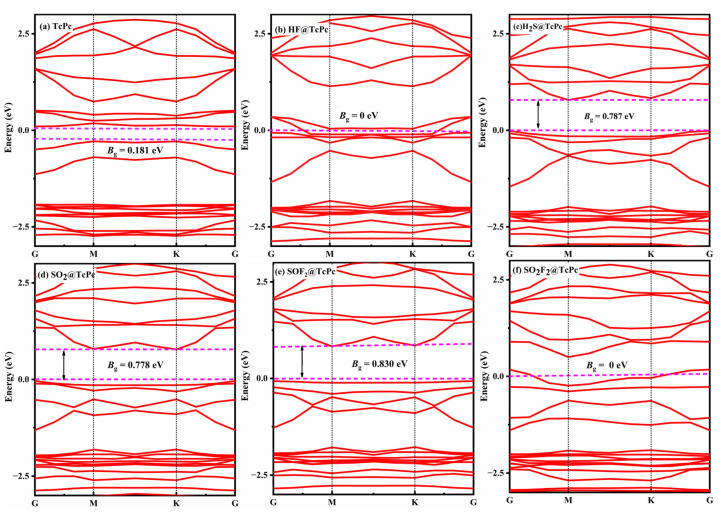
Band structure of various adsorption systems. (**a**) TcPc, (**b**) HF@TcPc, (**c**) H_2_S@TcPc, (**d**) SO_2_@TcPc, (**e**) SOF_2_@TcPc, and (**f**) SO_2_F_2_@TcPc. The Fermi energy is set as zero, and the space between the dashed colored lines represents the bandgap.

The adsorption of SF_6_ decomposition products induces a change in the conductivity (*σ*) of the substrate, which can serve as an indicator for the sensitivity of the material. The conductivity is determined by the bandgap (*B*_g_), as defined by the following formula:

(1) $$\sigma \propto A\exp\left(-B_{g}/2K_{B}T\right)$$

where A is a constant, while *K*_B_ and *T* represent the Boltzmann constant (8.62 × 10^−5^ eV/K) and absolute temperature, respectively. Based on this formula, a greater variance of bandgap before and after gas adsorption indicates a higher sensitivity for a material. The *B*_g_ of the TcPc nanosheet with and without gas adsorption is illustrated in Figure 12. It is evident that all of the systems exhibit semiconducting properties with a bandgap ranging from 0 eV to 0.830 eV. Compared to the *B*_g_ of pristine TcPc, the adsorption of HF and SO_2_F_2_ leads to a negligible change, indicating that the TcPc monolayer is less sensitive to these two gases. However, upon adsorption of H_2_S, SO_2_, and SOF_2_, the *B*_g_ of TcPc significantly increases from 0.181 eV to 0.787 eV, 0.778 eV, and 0.830 eV, respectively. This demonstrates its exceptional sensitivity to these gases, positioning it as a promising resistance-type gas sensor for detecting H_2_S, SO_2_, and SOF_2_.

**Figure 13:**
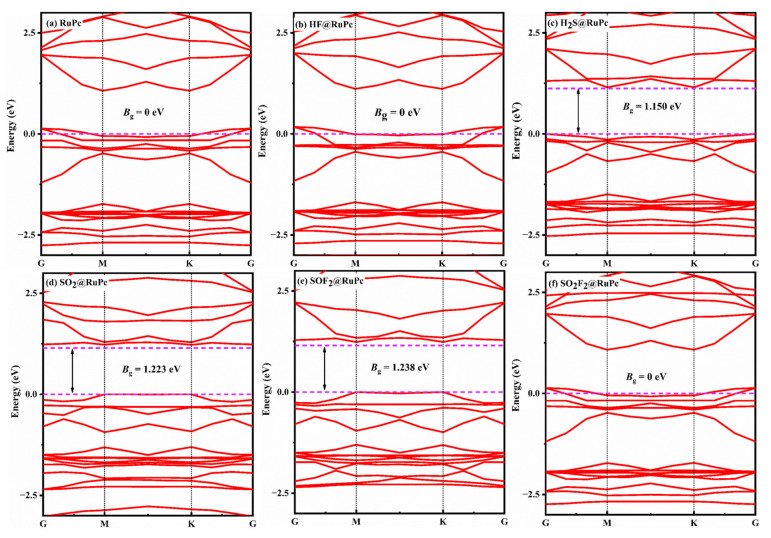
Band structure of various adsorption systems. (**a**) RuPc, (**b**) HF@RuPc, (**c**) H_2_S@RuPc, (**d**) SO_2_@RuPc, (**e**) SOF_2_@RuPc, and (**f**) SO_2_F_2_@RuPc. The Fermi energy is set as zero, and the space between the dashed colored lines represents the bandgap.

Figure 13 illustrates the band structures of various gas@RuPc adsorption systems. For the clean RuPc monolayer, it exhibits a zero bandgap with semimetal characteristics (Figure 13a). Upon the adsorption of H_2_S, SO_2_, and SOF_2_, the bandgap of RuPc is increased to 1.150 eV, 1.223 eV, and 1.238 eV, respectively. The significant changes in the bandgap of a RuPc monolayer suggest a transition from its semi-metallic properties to a semiconductor behavior. In other words, the RuPc monolayer demonstrates outstanding sensitivity towards H_2_S, SO_2_, and SOF_2_ molecules.

The work function is a crucial property for investigating adsorption performance. It signifies the strength of electron binding within a metal. A higher work function indicates a lower likelihood of electron emission from the metal. Therefore, assessing the sensitivity of a TMPc monolayer can be also achieved by analyzing the change in work function (*φ*) before and after gas adsorption, as defined by [53,54]:

(2) $$\varphi =E_{\mathrm{vacuum}}-E_{\mathrm{fermi}}$$

where *E*_vacuum_ and *E*_fermi_ represent the vacuum level and Fermi level of the TMPc monolayer after gas adsorption.

**Figure 14:**
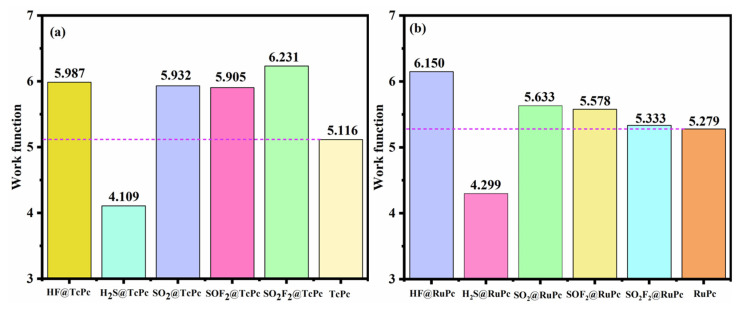
Work function of (**a**) TcPc and (**b**) RuPc monolayers with and without SF_6_-decomposed gas adsorption. The dashed colored lines represent the work function values of pristine TcPc and RuPc monolayer, respectively.

As depicted in Figure 14a, significant changes in the work function of TcPc are observed after the adsorption of SF_6_-decomposed gases. Comparing it to the *φ* of pristine TcPc (5.116 eV), the *φ* increases to 5.987 eV, 5.932 eV, 5.905 eV, and 6.231 eV after the adsorption of HF, SO_2_, SOF_2_, and SO_2_F_2_, respectively. Notably, the work function of the TcPc monolayer drops to 4.109 eV after H_2_S adsorption, highlighting the excellent gas-sensing capabilities of TcPc. From Figure 14b, one can find the significant alterations in the work function of RuPc upon the adsorption of SF_6_-decomposed gases. Compared to clean RuPc (*φ* = 5.279 eV), a minor increase in *φ* is observed after HF, SO_2_, SOF_2_, and SO_2_F_2_ adsorption. However, the work function of RuPc decreases significantly to 4.299 eV after H_2_S adsorption. Consequently, it can be concluded that RuPc also serves as an excellent gas sensor.

**Figure 15:**
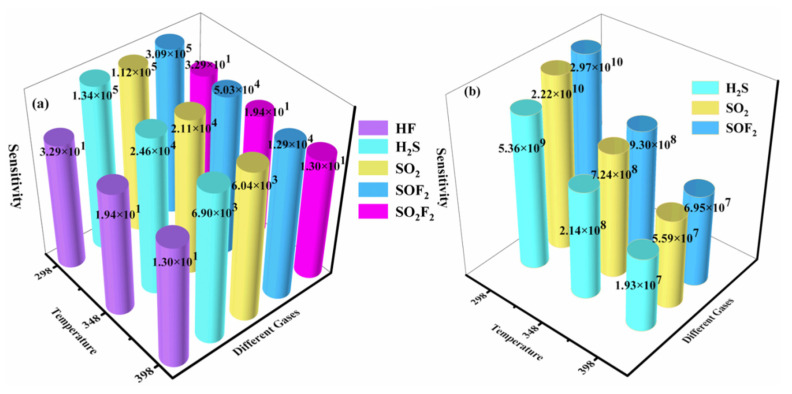
Sensitivity of (**a**) TcPc and (**b**) RuPc monolayers toward the SF_6_-decomposed gases at various temperatures.

The sensitivity of a gas-sensing material can be quantitatively assessed by measuring the resistance change of TMPc before and after gas adsorption. As is widely recognized, the conductivity of a substance is inversely proportional to its resistance. If the conductivity is recorded, the sensitivity of the sensor can be determined by the following equation [55]:

(3) $$S=(\frac{1}{\sigma_{\mathrm{TMPc}/\mathrm{gas}}}-\frac{1}{\sigma_{\mathrm{TMPc}}})/\frac{1}{\sigma_{\mathrm{TMPc}}}$$

where *σ*_TMPc/gas_ and *σ*_TMPc_ are the electrical conductivity of TMPc monolayer after and before adsorption, respectively. Figure 15 gives the sensitivity of five gas molecules on the TcPc and RuPc monolayers. As shown in Figure 15a, the sensitivity of the TcPc monolayer towards each gas gradually decreases as the temperature ranges from 298 K to 398 K, which is consistent with the sensitivity of the Rh-doped h-BN monolayer to SF_6_ decomposition gas [55]. Moreover, the TcPc monolayer exhibits the highest sensitivity to SOF_2_ at each temperature, followed by H_2_S, SO_2_, and SO_2_F_2_. Correspondingly, the TcPc monolayer exhibits outstanding sensitivity in detecting SO_2_, H_2_S, and SOF_2_ at its operating temperature. As illustrated in Figure 15b, it is evident that RuPc’s sensitivity to SF_6_ decomposition byproducts (H_2_S, SO_2_, and SOF_2_) at room temperature falls within the range of (5.36~29.7) × 10^9^, significantly exceeding the sensitivity towards SF_6_ alone (approximately 10^3^), and there is a slight decrease in sensitivity as the operating temperature rises. However, the sensitivity of H_2_S, SO_2_, and SOF_2_ at high temperature (398 K) is determined to be within the range of (1.93~6.95) × 10^7^, surpassing the sensitivity of Rh-BN towards SF_6_-decomposing species [55]. The exceptional sensitivity of the RuPc monolayer enables it to possess the precise ability to detect SF_6_ decomposition gases, underlining its potential for gas-sensing applications. In summary, both TcPc and RuPc monolayers exhibit remarkable capability in detecting these SF_6_-decomposing species.

## 3. Calculation Method and Details

In this work, the spin calculations were performed using the DMol^3^ quantum chemistry module [56] based on the density functional theory (DFT) method, as implemented in the Material Studio software package. The exchange–correlation among electrons is computed using the Perdew–Burke–Ernzerhof (PBE) function within the framework of general gradient approximation (GGA), owing to its superior computational accuracy [57]. To enhance the comprehension of the van der Waals force and long-range interactions, we employed the DFT-D method (Grimme custom) [58]. The double numerical plus polarization (DNP) [59] atomic orbital basis set was employed to ensure computational accuracy, while the DFT semi-core pseudopotential (DSPP) [60] was utilized to account for relativistic effects. Moreover, a real-space global cutoff radius of 5.2 Å was employed, and the Monkhorst–Pack scheme [61] was utilized to select k-points of 6 × 6 × 1 (12 × 12 × 1) meshes for geometry optimization (electronic property calculations). A vacuum space with a thickness of 20 Å was selected in the z-direction to mitigate interactions between neighboring clusters and to avoid interlayer interactions due to periodic boundary conditions [62]. The energy convergence, maximum displacement, and maximum force were respectively set as 1.0 × 10^−5^ Ha, 5 × 10^−3^ Å, and 0.002 Ha/Å.

In order to evaluate the stability of the TMPc monolayers, the binding energy (*E*_bin_) of the TM-doped Pc sheet was determined by [12,47]:

(4) $$E_{\mathrm{bin}}=E_{\mathrm{TM}+\mathrm{Pc}}-E_{\mathrm{Pc}}-E_{\mathrm{TM}}$$

where *E*_TM+Pc_ and *E*_Pc_ are the total energies of TMPc and pristine Pc monolayers, and *E*_TM_ is a single metal atom obtained by calculating the energy of corresponding bulk (*E*_TM(bulk)_). In addition, the cohesive energy (*E*_coh_) was also calculated to explore the aggregation possibility of TM atoms in the Pc monolayer. *E*_coh_ = (*E*_TM(bulk)_ − *E*_iso-TM_)/n, where *E*_iso-TM_ represents the energy of an isolated TM atom, and n is the number of TM atoms in bulk.

To quantitatively evaluate the interaction strength between SF_6_ decomposition products and the pristine H_2_Pc and TMPc monolayers, the adsorption energy (*E*_ads_) was calculated by:

(5) $$E_{\mathrm{ads}}=E_{\mathrm{gas}+\mathrm{sur}}-E_{\mathrm{gas}}-E_{\mathrm{sur}}$$

where *E*_gas+sur_ is the total energy of TMPc with the adsorbed SF_6_-decomposed gas molecules, and *E*_sur_ and *E*_gas_ are the total energy of the clean H_2_Pc/TMPc monolayer and particular decomposed gas molecule, respectively. When the *E*_ads_ is negative, the adsorption process becomes spontaneous and releases heat, and a larger absolute value of *E*_ads_ means stronger adsorption strength. The electron transfer (Δ*Q*) from the substrate to SF_6_ decomposition products based on the Hirshfeld charge can be determined by:

(6) $$Q_{t}=Q_{\mathrm{adsorbed}}-Q_{\mathrm{isolated}}$$

The *Q*_isolated_ and *Q*_adsorbed_ represent the charge of SF_6_ decomposition products before and after adsorption, respectively. A negative (positive) value of Δ*Q* indicates that the SF_6_ decomposition product’s gas molecule gains (loses) electrons. In addition, the charge density difference (CDD, Δ*ρ*) of different adsorption systems can be obtained by:

(7) $$\Delta \rho =\rho_{\mathrm{slab}+\mathrm{gas}}-\rho_{\mathrm{slab}}-\rho_{\mathrm{gas}}$$

where *ρ*_slab+gas_, *ρ*_slab_, and *ρ*_gas_ represent the electron density of the TMPc monolayer with gas adsorption, a clean TMPc monolayer, and isolated SF_6_ decomposition products, respectively.

## 4. Conclusions

In this work, first-principles calculations were utilized to investigate the adsorption behaviors of SF_6_-decomposed species (HF, SO_2_, H_2_S, SOF_2_, and SO_2_F_2_) on the intrinsic and Tc/Ru-doped H_2_Pc monolayers, aiming to find a potential Pc-based gas-sensing material for the detection or scavenging of the above five gas molecules. The main conclusions are summarized as follows:

(1) TcPc and RuPc monolayers exhibit a semi-metallic property, and the strong hybridizations between the Tc/Ru-d orbital and N4 of Pc further demonstrate their high structural stability.
(2) The TcPc monolayer exhibits a strong affinity towards H_2_S, SO_2_, SOF_2_, and SO_2_F_2_ due to the robust orbital hybridization between the Tc-d orbitals and S-sp orbitals of these gases.
(3) The RuPc nanosheet exhibits a remarkable ability to capture H_2_S, SO_2_, and SOF_2_ molecules, primarily owing to the robust orbital hybridizations between the Ru-d orbitals and the S-sp orbitals of these gases. Therefore, the RuPc nanosheet holds significant promise as a scavenger for H_2_S, SO_2_, and SOF_2_ molecules.
(4) The adsorption of H_2_S, SO_2_, and SOF_2_ induces significant changes in the bandgap and work function of the TcPc and RuPc monolayers, highlighting the strong sensitivity of these monolayers to H_2_S, SO_2_, and SOF_2_ molecules.

Overall, the bare H_2_Pc monolayer is not a potential gas-sensing material for SF_6_-decomposed species. However, the TMPc (TM = Tc and Ru) monolayer shows promise as a potential material for the gas detection or scavenging of SF_6_-decomposed species, owing to its enhanced gas capture ability and heightened sensitivity. These theoretical results can provide certain guidance for subsequent related experimental research.

## Figures and Tables

**Table 1:** Preferential adsorption orientation (Orientation), adsorption energy (*E*_ads_), adsorption distances (*D*), electron transfer (*Q*_t_), and bandgap (*B*_g_) of the most stable adsorption system, where gas@H_2_Pc is defined as the SF_6_ decomposition products adsorbed on the H_2_Pc substrate. When H_2_Pc does not adsorb gas, the band gap is 1.120 eV.

Adsorption System	Orientation	*E*_ads_/eV	*Q*_t_/e	*B*_g_/eV
HF@H_2_Pc	H-end	−0.15	−0.08	1.106
H_2_S@H_2_Pc	S-end	−0.14	0.027	1.125
SO_2_@H_2_Pc	S-end	−0.33	−0.054	1.092
SOF_2_@H_2_Pc	S-end	−0.27	−0.309	1.106
SO_2_F_2_@H_2_Pc	S-end	−0.24	0.043	1.121

**Table 2:** Preferential adsorption orientation (Orientation), adsorption energy (*E*_ads_), adsorption distance (*D*), electron transfer (*Q*_t_), and bandgap (*B*_g_) of the most stable adsorption system, where gas@TcPc is defined as the SF_6_ decomposition products adsorbed on the TcPc substrate.

Adsorption System	Orientation	*E*_ads_/eV	*D*/Å	*Q*_t_/e	*B*_g_/eV
HF@TcPc	H-end	−0.23	2.368	−0.140	0.000
H_2_S@TcPc	S-end	−1.43	2.308	0.298	0.787
SO_2_@TcPc	S-end	−1.97	2.160	−0.099	0.778
SOF_2_@TcPc	S-end	−1.78	2.132	−0.073	0.830
SO_2_F_2_@TcPc	S-end	−0.96	2.353	−0.256	0.000

**Table 3:** Preferential adsorption orientation (Orientation), adsorption energy (*E*_ads_), adsorption distance (*D*), the electron transfer (*Q*_t_), and bandgap (*B*_g_) of the most stable adsorption system, where gas@RuPc is defined as the SF_6_ decomposition products adsorbed on the RuPc substrate.

Adsorption System	Orientation	*E*_ads_/eV	*D*/Å	*Q*_t_/e	*B*_g_/eV
HF@RuPc	H-end	−0.28	2.268	−0.165	0.000
H_2_S@RuPc	S-end	−1.26	2.243	0.275	1.150
SO_2_@RuPc	S-end	−1.64	2.110	−0.071	1.223
SOF_2_@RuPc	S-end	−1.53	2.088	−0.039	1.238
SO_2_F_2_@RuPc	S-end	−0.33	3.411	0.003	0.000

## Data Availability

The data used in this study are contained within the article.
